# Difference in immune response in vaccinated and unvaccinated Swedish individuals after the 2009 influenza pandemic

**DOI:** 10.1186/1471-2334-14-319

**Published:** 2014-06-11

**Authors:** Isabelle Magalhaes, Mikael Eriksson, Charlotte Linde, Rashid Muhammad, Lalit Rane, Aditya Ambati, Rebecca Axelsson-Robertson, Bahareh Khalaj, Nancy Alvarez-Corrales, Giulia Lapini, Emanuele Montomoli, Annika Linde, Nancy L Pedersen, Markus Maeurer

**Affiliations:** 1 Center for allogeneic stem cell transplantation, Karolinska University Hospital, Stockholm, Sweden; 2 Department of Laboratory Medicine, Division of Therapeutic Immunology, Karolinska Institute, Stockholm, Sweden; 3 Department of Medical Epidemiology and Biostatistics, Karolinska Institute, Stockholm, Sweden; 4 Microbiology, Tumor and Cell Biology, MTB, Karolinska Institutet, Stockholm, Sweden; 5 Department of Physiopathology, Molecular Epidemiology Research Division, Experimental Medicine and Public Health, University of Siena, Siena, Italy; 6 Swedish Institute for Communicable Disease Control Stockholm, Smittskyddsinstitutet, Solna, Sweden; 7 Therapeutic Immunology, F79, LabMed, Hälsovägen, Karolinska University Hospital Huddinge, SE-14186 Huddinge, Sweden

**Keywords:** T-cells, H1N1, Immune protection, Flu antigens, Vaccine take, Epidemiology, Influenza, Pandemic

## Abstract

**Background:**

Previous exposures to flu and subsequent immune responses may impact on 2009/2010 pandemic flu vaccine responses and clinical symptoms upon infection with the 2009 pandemic H1N1 influenza strain. Qualitative and quantitative differences in humoral and cellular immune responses associated with the flu vaccination in 2009/2010 (pandemic H1N1 vaccine) and natural infection have not yet been described in detail. We designed a longitudinal study to examine influenza- (flu-) specific immune responses and the association between pre-existing flu responses, symptoms of influenza-like illness (ILI), impact of pandemic flu infection, and pandemic flu vaccination in a cohort of 2,040 individuals in Sweden in 2009–2010.

**Methods:**

Cellular flu-specific immune responses were assessed by whole-blood antigen stimulation assay, and humoral responses by a single radial hemolysis test.

**Results:**

Previous seasonal flu vaccination was associated with significantly lower flu-specific IFN-γ responses (using a whole-blood assay) at study entry. Pandemic flu vaccination induced long-lived T-cell responses (measured by IFN-γ production) to influenza A strains, influenza B strains, and the matrix (M1) antigen. In contrast, individuals with pandemic flu infection (PCR positive) exhibited increased flu-specific T-cell responses shortly after onset of ILI symptoms but the immune response decreased after the flu season (spring 2010). We identified non-pandemic-flu vaccinated participants without ILI symptoms who showed an IFN-γ production profile similar to pandemic-flu infected participants, suggesting exposure without experiencing clinical symptoms.

**Conclusions:**

Strong and long-lived flu-M1 specific immune responses, defined by IFN-γ production, in individuals after vaccination suggest that M1-responses may contribute to protective cellular immune responses. Silent flu infections appeared to be frequent in 2009/2010. The pandemic flu vaccine induced qualitatively and quantitatively different humoral and cellular immune responses as compared to infection with the 2009 H1N1 pandemic H1N1 influenza strain.

## Background

The 2009 H1N1 pandemic H1N1 influenza (flu) A (pdmH1N1) was first reported in Mexico and California [1]. As the virus spread globally, it became evident that the infection was of moderate severity with a broad clinical spectrum of symptoms [2]. Early data from Mexico suggested that pdmH1N1 may cause severe respiratory illness in otherwise healthy young and middle-aged people [3]. It is now estimated that in the United States pdmH1N1 caused higher rates of hospitalizations and deaths in children and adults 18–64 years of age than the flu of the previous season, but lower rates of clinical events in adults over 65 years of age [4]. This finding supports the notion that previous exposures to H1N1 in older individuals provides higher cross-protective immune responses than in younger individuals [5]. In the autumn of 2009, pdmH1N1 vaccines (pdm vaccines) were available across the globe, it was estimated that pdmH1N1 vaccination prevented 4,000–10,000 hospitalizations and 200–500 deaths in the US [6].

There are several unanswered questions concerning H1N1 flu infection and vaccination. Firstly, the impact of previous immune responses induced by flu vaccinations or by flu exposures on the ability to mount new anti-flu immune responses [7-9]. It is therefore of interest to map the adaptive humoral and cellular immune response prior to vaccination and prior to the onset of flu symptoms. Secondly, the differential quality and quantity of humoral and cellular immune responses directed against flu targets, induced either by flu vaccination or by flu infection, has not been well defined. Thirdly, several reports have discussed a ‘silent infection’ with H1N1 (in non-vaccinated participants), yet the real extent of such a silent infection is difficult to determine—in part due to cross-reacting antibodies (Abs) (from previous exposures or vaccines) [10]. Antibodies, as well as CD4+ and cytotoxic CD8+ T-cell immune responses, play a critical role in the host defense against flu [11,12]. Increased cellular reactivity to flu, defined by IFN-γ production—a key cytokine in the pro-inflammatory response to flu—in participants who have not experienced influenza-like (ILI) symptoms, has not been determined up to now, this may help to identify silent flu infection.

In order to address these questions, we designed a prospective study of participants living in the Stockholm area in the context of the LifeGene (LG) project [13] to map in detail the breadth of the cellular immune responses *prior* to the onset of ILI symptoms and prior to vaccination with the pdm flu vaccine to study i) pre-existing immune responses, ii) the association of ILI symptoms with vaccination, iii) cellular immune reactivity during the flu season 2009–2010, directed against a broad panel of flu pathogens. This prospective study was designed to decipher differences in immune responses induced by infection with pdmH1N1 and the pdm vaccine, and this is the first report to describe in an unbiased fashion humoral and cellular immune responses during the pdmH1N1 infection in 2009–2010 in Sweden.

## Methods

### Study participants

2,040 study participants from Stockholm (Sweden) entered the baseline step of the ILI study in September 2009, a part of the LG project [13]. For details see the Additional file 1: Text Material S1. Serum and heparin blood samples were drawn at study entry and after the flu season in the spring of 2010. Prepaid envelopes and nasal swabs were provided to participants after they had received instructions at the LifeGene study centres before study entry. Participants contacted the LG centres at the onset of ILI symptoms, filled out a questionnaire and mailed the (viral) swab for PCR analysis as described in detail in the supplementary data sets. If swabs tested positive for flu or coronavirus RNA, a home visit was payed during which an additional nasal swab and a blood sample from the index study participant and the household members was obtained. (The mean time between the first swab and the home visit was 2.5 weeks). The study was approved by the Ethics committee Stockholm south review board (DN 2009/1183-31) and each study participant provided informed consent. Swedish residents were offered the pdm vaccine Pandemrix (GSK) containing A/ H1N1/California/7/2009 (with AS03 adjuvant, i.e. DL-α-tocopherol, squalene and polysorbate).

### Functional T-cell assay - whole-blood antigen stimulation assay

Forty μL of heparinised blood was diluted 1:5 in RPMI 1640 with L-glutamine (2 mM), penicillin (100 IU/mL) and streptomycin (10 mg/mL), and processed as described in detail in the supplementary data section (Additional file 1). The whole-blood antigen (WBA) stimulation assay measures the ‘net’ IFN-γ production elaborated by CD4+ and CD8+ T-cells in whole blood and reflects the activity of memory T-cell responses. We tested several flu antigens from previous monovalent flu vaccines, composed of haemagglutinin (HA) and neuraminidase (N) from flu A or B strains, for the capacity to elicit IFN-γ responses in T-cells: A/H1N1/Brisbane/59/2007, A/H1N1/Solomon Islands/3/2006, A/H3N2/Uruguay/716/2007, A/H3N2/Wisconsin/67/2005, B/Malaysia/2506/2004, B/Florida/4/2006, or A/H5N1/Vietnam/1203/2004, all provided by Baxter Innovations GmbH (Vienna, Austria). They were used at a final concentration of 2.5 μg/mL HA. We also tested the flu matrix antigens M1 and M2 present as peptides, and used at a final concentration of 1 μg/mL. As control, we used the CFP-10 antigen from *Mycobacterium tuberculosis*. We could not include the A/H1N1/California/7/2009 antigens in the cellular assays, since the test plates had to be prepared in advance due to quality control issues to ensure batch-to-batch consistency; the pdmH1N1 antigen preparation was not yet available in the summer of 2009 and the assay worked only with freshly obtained heparinised blood. In contrast to the cellular assay, Ab titres could be tested retrospectively.

### Swab processing and PCR analysis

If study participants experienced symptoms of ILI, they performed a nasal swab to be tested for 22 viral targets (Additional file 1: Table S1) as described [14]. Swab processing and PCR are described in detail in the supplementary data section (Additional file 1: Text Material S1). This step was taken to link ILI symptoms with the detection of defined viral pathogens. In addition, if the PCR tested positive for H1N1, a nurse visited the participant 14 days (on average) after onset of symptoms in order to obtain a blood sample, which allowed study of the humoral and cellular immune response early after infection. This was also performed for participants infected with coronavirus as a control group.

### Ab determination - single radial haemolysis test

Sera, collected at study entry and after the flu season in the spring of 2010, and during a home visit in case of a positive H1N1 or coronavirus PCR (detected from a nasal swab), were stored at −80°C until testing. Single radial haemolysis (SRH) test against A/H1N1/California/07/2009 was performed at the Department of Physiopathology, Experimental Medicine and Public Health of the University of Siena according to procedures described in detail elsewhere [15]. Diameters of the haemolysis area for each serum tested were measured. Sera with areas of haemolysis equal to or higher than 4 mm^2^ but lower than 25 mm^2^ were considered to indicate seropositivity but not protection; haemolysis areas equal to or higher than 25 mm^2^ were considered to indicate seroprotection.

### Statistical analysis

Statistical analysis was performed on (i) the IFN-γ concentrations as a cellular response to flu and non-flu antigens, (ii) the PCR-based virus detection, (iii) the SRH Ab data, and (iv) the questionnaire data including symptoms reported in a web-based questionnaire by the study participants during and after the flu season. Data were analysed using SAS 9.2 software (SAS Institute). Descriptive statistics were used and Spearman analysis for independence was performed to verify integrity of data. Significance analysis was performed using the Pearson χ^2^-test, Student’s t-test, and the Wilcoxon-Mann–Whitney two-sample rank-sum test. Multivariate operations, including operations on stratified variable dependencies and cluster analysis (using the VARCLUS procedure), were used in the analysis based on the combined dataset constituents, i.e. IFN-γ, PCR, antigen-specific Abs, symptoms, and vaccination. For each analysis, the nature of the statistical test used is indicated in the corresponding figure legend and table.

## Results

### Study cohort

**Figure 1:**
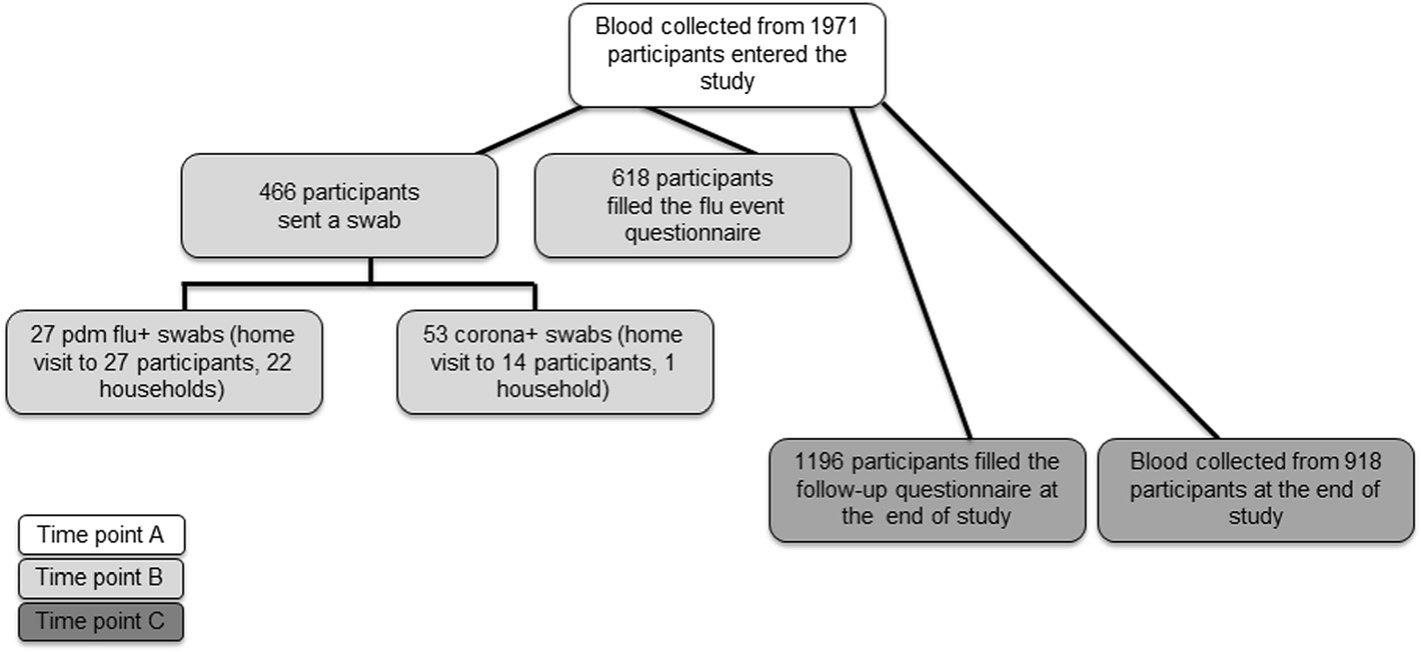
**Overview of the ILI study participants.** 1,971 study participants entered the study in the winter of 2009 (time point A); 67 samples were collected from study participants and household members during home visits triggered by an H1N1+ or coronavirus + PCR (detected in nasal swabs sent at time point B); 918 study participants returned for the follow-up sampling in the spring of 2010 (time point C).

After quality control (i.e. exclusion of samples without identification), the analysis group consisted of 1,971 participants: 1,807 adults aged between 18 and 65 years (median age 36, 53% women), 155 adults over 65 (median age 71 years, 65% women), and 9 children with a median age of 8 years. The baseline step (autumn/winter 2009) is designated time point ‘A’, and the event step (autumn/winter 2009) is designated time point ‘B’. Altogether, 466 study participants mailed a swab for viral pathogen analysis and 618 participants filed a self-report (flu event questionnaire). For 41 study participants, a positive H1N1 or coronavirus PCR (for controls) triggered a home visit from a nurse to study the immune response at an early time point. The final step (spring 2010) is designated time point ‘C’, where 918 study participants (46.6% of the initial number of participants) again provided a blood sample (see overview of the study in Figure 1). This allowed us to study the humoral and cellular immune responses directed against flu—and also against control antigens—in an unbiased way.

PdmH1N1 ‘swine flu’ RNA could be detected in 27 swabs (24 from study participants and 3 from household members), and coronavirus RNA could be detected in 53 swabs (from 51 study participants and 2 household members) (see overview of the swabs analysis in Additional file 1: Table S2).

### The initial set-point of A/H1N1/California/7/2009-specific antibodies and IFN-γ production

Study participants reported their flu vaccination status in the web questionnaires: 25.3% of study participants had received at least a single previous seasonal flu vaccination between 2006 and 2009; 63.1% (n = 755) of the study participants who responded to the follow-up questionnaire in the spring received the pdm flu vaccine in 2009–2010.

We evaluated the impact of previous flu vaccinations on IFN-γ production and A/H1N1/California/7/2009-specific Ab levels measured by SRH at study entry (Table 1). The analysis showed significantly lower levels of IFN-γ production in response to A/H1N1/Brisbane/59/2007, A/H1N1/Solomon Islands/3/2006 and B/Florida/4/2006 in blood from study participants who had previously received seasonal flu vaccination (during the period 2006 to spring 2009) and who did not receive the pdm flu vaccine, than in those without any seasonal flu (or pdm) vaccination during the same period (p ≤ 0.04). We could not detect any significant differences in levels of A/H1N1/California/7/2009-specific Abs between vaccinated and unvaccinated participants. Notably, both groups were considered seropositive at SRH > 4 mm^2^, but below protective levels (SRH < 25 mm^2^), at time point A (Table 1). Additionally, for time point A, we measured by the flu hemagglutination inhibition assay the presence of Abs directed against: A/H1N1/California/7/2009, A/H1N1/Brisbane/59/2007, A/H1N1/Solomon Islands/3/2006, A/H3N2/Uruguay/716/2007 and A/H5N1/Vietnam/1203/2004 in 7 participants (above 50 years of age) who had received previous seasonal flu vaccination, and 7 age-matched participants who had not received previous seasonal flu vaccination. Interestingly, 2 and 3 participants (out of 7 participants who did not receive seasonal flu vaccination) showed measurable levels of Abs specific for A/H1N1/Brisbane/59/2007 and A/H1N1/Solomon Islands/3/2006, respectively, but only 1 participant (out 7 participants who had received previous seasonal flu vaccination) showed measurable levels of Abs specific for A/H1N1/Solomon Islands/3/2006 (data not shown).

**Table 1:** Mean values of A/H1N1/California/7/2009-specific Abs (measured by SRH) and IFN-γ production (in whole-blood assay) before (A) and after (C) the 2009–2010 flu season, in blood from study participants with our without seasonal flu vaccination (2006–2009)

**Time point**	**A**		**C**		**A**	**C**
	**2006-2009 seasonal flu vacc.**					
	**Vacc.**	**Not vacc.**	**Vacc.**	**Not vacc.**	**V/NV**^**1**x^**-diff.**	
	**SRH (mm**^**2**^**), geometric mean (C.I.)**				***p-value***	
**A/H1N1/California**	18.3 (14.2–23.7)	19.6 (17.5–22.0)	19.1 (15.5–23.6)	21.2 (19.2–23.4)	*0.5*	*0.5*
	**IFN-γ (pg/mL), mean**					
**A/H1N1/Brisbane**	99	144	168	172	***0.04***	*0.6*
**A/H1N1/Solomon**	85	122	152	153	***0.03***	*0.3*
**A/H3N2/Uruguay**	137	190	221	245	*0.12*	*0.3*
**A/H3N2/Wisconsin**	162	206	226	261	*0.3*	*0.2*
**A/H5N1/Vietnam**	325	361	377	373	*0.4*	*0.9*
**B/Florida**	204	265	260	289	***0.04***	*0.4*
**B/Malaysia**	154	173	164	212	*0.14*	*0.2*
**M1**	24	37	35	33	*0.14*	*0.14*
**M2**	18	18	19	21	*0.7*	*0.7*
**CFP-10**	20	22	18	23	*0.4*	*0.3*
**PHA**	518	512	545	544	*0.6*	*0.5*
**N**	57	175	57	175		
**Mean age, years**	50	38	50	38	*<0.001*	*<0.001*

The Wilcoxon-Mann–Whitney rank-sum test was used to calculate p-values. ^1^V/NV: vaccinated vs. unvaccinated. Listed are Ab responses (top panel, directed against A/H1N1 California, mean and confidence interval) and IFN-γ responses directed against a broad panel of flu targets (designated the flu A or flu B strains), and against matrix proteins M1 and M2. CFP10 served as a control. PHA was the positive control stimulus. P values below 0.05 are indicated in bold.

In contrast to the differences observed at entry into the study (time point A), no differences were observed in IFN-γ production between study participants with or without previous seasonal flu vaccinations (Table 1) at time point C. At this time point, levels of A/H1N1/California/7/2009-specific Abs were below protective levels (SRH < 25 mm^2^) in both vaccinated and unvaccinated participants.

### The immunological footprint of pdm vaccination

We followed the study participants who gave blood at time points A and C and reported (in the spring of 2010) their 2009–2010 pandemic vaccination status. At time point A, the levels of A/H1N1/California/7/2009-specific Abs and IFN-γ production in response to the flu antigens were comparable between participants who received (after time point A) the pdm flu vaccine and individuals who chose not to receive the vaccine (seropositive at SRH > 4 mm^2^, but below protective levels (SRH < 25 mm^2^)).

At time point C, the levels of A/H1N1/California/7/2009-specific Abs were above protective levels (SRH ≥ 25 mm^2^) in pdm flu vaccinated participants, the Ab levels were significantly higher (p < 0.001) than in non-pmd flu vaccinated study participants. We also observed a statistically significant increase in IFN-γ production in response to all flu antigens (except for B/Malaysia/2506/2004), including the flu matrix antigen M1, in blood from study participants who received the pdm flu vaccine in the winter of 2009–2010 compared to the non-vaccinated study participants (p ≤ 0.04) (Table 2; note that M1 is not a designated component of the flu vaccine).

**Table 2:** Mean values of A/H1N1/California/7/2009-specific Abs (measured by SRH) and IFN-γ production in the whole-blood assay before (A) and after (C) the 2009/2010 flu season in study participants with our without pdm flu vaccination

**Time point**	**A**		**C**		**A**	**C**
	**pdm vaccination**					
	**Vacc.**	**Not vacc.**	**Vacc.**	**Not vacc.**	**V/NV**^**1**^**-diff.**	
	**SRH (mm**^**2**^**), geometric mean**					
**A/H1N1/California**	20.1 (18.5–21.8)	19.3 (17.3–21.4)	27.9 (26.4–29.5)	20.7 (18.9–22.6)	*0.3*	***<0.001***
	**IFN-γ (pg/mL), mean**					
**A/H1N1/Brisbane**	139	133	285	169	*0.3*	***<0.001***
**A/H1N1/Solomon**	119	113	246	151	*0.2*	***<0.001***
**A/H3N2/Uruguay**	194	177	304	238	*0.2*	***<0.001***
**A/H3N2/Wisconsin**	203	195	301	251	*0.6*	***<0.01***
**A/H5N1/Vietnam**	346	352	423	373	*0.6*	***<0.01***
**B/Florida**	221	250	245	283	*0.1*	***0.04***
**B/Malaysia**	145	168	167	200	*0.15*	*0.07*
**M1**	33	34	63	34	*0.8*	***<0.01***
**M2**	18	18	20	20	*0.4*	*0.8*
**CFP-10**	21	21	24	22	*0.7*	*0.2*
**PHA**	501	513	506	545	*0.13*	*0.02*
**N**	437	232	437	232		
**Mean age, years**	47	41	47	41	*<0.001*	*<0.001*

The Wilcoxon-Mann–Whitney rank-sum test was used to calculate p-values. ^1^V/NV: vaccinated vs. unvaccinated. Listed are Ab responses (top panel, directed against A/H1N1 California, mean and confidence interval) and IFN-γ responses directed against a broad panel of flu targets (designated the flu A or flu B strains), and against matrix proteins M1 and M2. CFP10 served as control. PHA was the positive control stimulus. P values below 0.05 are indicated in bold.

### Experience of ILI symptoms and the effect of pdm flu vaccination

We examined whether ILI symptoms segregated with the flu vaccination records. 58.0% of study participants who were vaccinated also reported the experience of ILI symptoms. 65 study participants who received the pdm flu vaccine and filed their ILI symptoms report later during the flu season (i.e. after December 2009, and after they had received the pdm flu vaccine) experienced significantly more symptoms than the 170 study participants who did not receive the pdm flu vaccine (and also reported their ILI symptoms after December 2009). Vaccinated study participants reported 4 ILI symptoms rather than 3 symptoms on average, i.e. nasal discharge (p = 0.02), fatigue (p = 0.04), cough (p = 0.01), sneezing (p = 0.04) and chills (p = 0.05) (Table 3). The 45 study participants who received the pdm flu vaccine and mailed a swab later showed less frequent detection (6/45) of coronavirus RNA (p = 0.03), but showed a higher percentage (14/45) of rhinovirus RNA (p = 0.01) in their nasal swabs as compared to the 139 study participants who chose not to get vaccinated but mailed a swab due to symptoms of ILI (44/139).

**Table 3:** Reported distribution of symptoms in study participants with or without pdm flu vaccination

**Symptoms**	**Vaccinated**		**Not vaccinated**		**Difference**
	**N**	**%**	**N**	**%**	***p-value***
**Nasal discharge**	43	15.1	84	14.8	***0.02***
**Fatigue**	36	12.6	68	12.0	***0.04***
**Cough**	35	12.3	61	10.7	***0.01***
**Headache**	28	9.8	62	10.9	*0.4*
**Sneezing**	35	12.3	66	11.6	*0.04*
**Sore throat**	23	8.1	65	11.4	*0.7*
**Muscle aches or joint pain**	26	9.1	48	8.4	*0.08*
**Chills**	22	7.7	36	6.3	***0.05***
**Other symptoms**	9	3.2	20	3.5	*0.7*
**Fever**	9	3.2	20	3.5	*0.7*
**Ear pain**	7	2.5	21	3.7	*0.7*
**Chest discomfort**	6	2.1	9	1.6	*0.3*
**Diarrhea**	3	1.1	5	0.9	*0.5*
**Itch**	2	0.7	4	0.7	*0.8*
**Vomiting**	1	0.4	0	0.0	*0.1*
**Symptoms N/%**	285	100	569	100	
**N**	65	65	170	170	

Note the differential symptom presentation in participants vaccinated or not vaccinated against pdmH1N1. P values below 0.05 are indicated in bold.

### IFN-γ production profiles and A/H1N1/California/7/2009-specific Abs showed differences in unvaccinated and vaccinated study participants depending on their ILI symptom status

We analysed the immune status from study participants who did not report any symptoms of ILI since they were either (i) not exposed to pathogens leading to ILI symptoms, (ii) they may have been protected due to pre-existing anti-flu directed immune responses or pdm flu vaccination.

We show in Table 4 significant differences (time point C versus time point A) in IFN-γ production and A/H1N1/California/7/2009-specific Abs in blood samples from study participants who received the pdm flu vaccine and reported *no ILI symptoms* versus blood samples from unvaccinated study participants (reporting no ILI symptoms). At time point C (i.e. after the flu season) we identified higher levels of A/H1N1/California/7/2009-specific Abs (p < 0.001) and IFN-γ production in response to A/H5N1/Vietnam/1203/2004 (p = 0.03), to A/H1N1/Brisbane/59/2007, to A/H1N1/Solomon Islands/3/2006 (p < 0.001) as well as to the M1 matrix antigen (p < 0.01) in study participants who received the pdm flu vaccine.

**Table 4:** Mean values of A/H1N1/California/7/2009-specific Abs and IFN-γ production in study participants with or without pdmH1N1 vaccination who did not report any symptoms of ILI during the 2009–2010 flu season

	**Not vaccinated**			**Vaccinated**			**Difference**	
	**A**	**C**	**C-A**	**A**	**C**	**C-A**	**C-A V-N**^**1**^	***p-value***
	**SRH (mm**^**2**^**), geometric mean**							
**A/H1N1/California**	20.9	23.3	2.4	18.8	28.6	9.8	7.4	***<0.001***
	**IFN-γ (pg/mL), mean**							
**A/H1N1/Brisbane**	134	164	30	101	234	134	105	***<0.01***
**A/H1N1/ Solomon**	117	142	25	60	190	130	105	***<0.001***
**A/H3N2/Uruguay**	167	232	64	148	256	109	37	*0.07*
**A/H3N2/Wisconsin**	172	243	71	145	253	108	37	*0.4*
**A/H5N1/Vietnam**	338	338	0	307	395	88	88	***0.03***
**B/Florida**	233	275	42	196	208	12	−27	*0.6*
**B/Malaysia**	155	197	42	125	141	15	−27	*0.3*
**M1**	32	29	−3	22	49	27	30	***<0.01***
**M2**	18	22	4	18	18	0	−4	*0.9*
**CFP-10**	19	18	−1	20	23	3	4	*0.2*
**PHA**	501	538	37	517	519	2	−35	*0.8*
**Mean IFN-γ diff.**	163	191	28	144	198	53	25	
**N**	94	94		120	120			

The Wilcoxon-Mann–Whitney rank-sum test was used to calculate the p-values (comparison of C-A between vaccinated and unvaccinated). ^1^V-N is vaccinated minus unvaccinated. Listed are Ab responses (top panel, directed against A/H1N1 California, mean and confidence interval) and IFN-γ responses directed against a broad panel of flu targets (designated the flu A or flu B strains), and against matrix proteins M1 and M2. CFP10 served as control. PHA was the positive control stimulus. P values below 0.05 are indicated in bold.

Next, we examined IFN-γ production and A/H1N1/California/7/2009-specific Abs in blood from study participants (vaccinated and non-vaccinated) who reported ILI symptoms (Table 5). We identified a slightly different immunological reactivity pattern as compared to the group of study participants who did not report any ILI symptoms at all (see above): (i) the increase (between time point C and time point A) in the mean value of A/H1N1/California/7/2009-specific Abs in unvaccinated and vaccinated study participants was not statistically significant, yet it reached protective levels (>25 mm^2^) at time point C for pdm flu vaccinated study participants (ii) vaccinated study participants showed a significant increase in IFN-γ production in response to A/H1N1/Brisbane/59/2007 (p < 0.001), to A/H3N2/Uruguay/716/2007, to A/H1N1/Solomon Islands/3/2006 (p ≤ 0.01), and to A/H3N2/Wisconsin/67/2005 (p = 0.02).

**Table 5:** Mean values of A/H1N1/California/7/2009-specific Abs and IFN-γ production in study participants with or without pdmH1N1 vaccination who reported symptoms of ILI during the 2009–2010 flu season

	**Not vaccinated**			**Vaccinated**			**Difference**	
	**A**	**C**	**C-A**	**A**	**C**	**C-A**	**C-A V-N**^**1**^	***P-value***
	**SRH (mm**^**2**^**), geometric mean**							
**A/H1N1/California**	15.3	19.5	4.2	18.8	28.0	9.2	5.0	*0.1*
	**IFN-γ (pg/mL)**							
**A/H1N1/Brisbane**	169	166	−2	113	296	183	185	***<0.001***
**A/H1N1/Solomon**	130	180	50	80	262	182	132	***<0.01***
**A/H3N2/Uruguay**	197	250	53	177	336	159	106	***0.01***
**A/H3N2/Wisconsin**	217	248	32	185	343	157	125	***0.02***
**A/H5N1/Vietnam**	330	374	44	310	403	93	49	*0.3*
**B/Florida**	254	276	22	177	263	85	63	*0.4*
**B/Malaysia**	173	203	30	108	208	101	71	*0.08*
**M1**	43	34	−10	31	62	31	41	*0.09*
**M2**	18	18	0	18	18	0	0	*0.3*
**CFP-10**	18	20	2	22	19	−3	−5	*0.7*
**PHA**	505	557	53	498	536	39	−14	*0.9*
**Mean IFN-γ diff.**	168	200	33	146	230	84	51	
**N**	49	49		47	47			

The Wilcoxon-Mann–Whitney rank-sum test was used to calculate the p-values (comparison of C-A between vaccinated and unvaccinated). ^1^V-N is vaccinated minus unvaccinated. Listed are Ab responses (top panel, directed against A/H1N1 California, mean and confidence interval) and IFN-γ responses directed against a broad panel of flu targets (designated the flu A or flu B strains), and against matrix proteins M1 and M2. CFP10 served as control. PHA was the positive control stimulus. P values below 0.05 are indicated in bold.

### The immunological profile of H1N1 infection

We analysed in greater detail the IFN-γ production and A/H1N1/California/7/2009-specific Abs from 21 study participants who tested positive for pdmH1N1 RNA and received a home visit (time point B) after the ILI symptom report (Table 6); biological material from participants who tested positive for coronavirus RNA served as controls. Blood from participants with a positive pdmH1N1 PCR (but not the coronavirus positive controls), showed significantly higher levels of A/H1N1/California/7/2009-specific Abs (p ≤ 0.001) at time point B and at time point C (as compared to time point A, i.e. before the flu season). Participants testing positive for pdmH1N1 RNA also showed significantly higher levels of IFN-γ production at time point B than at time point A (p ≤ 0.01) in response to all flu antigens including M1, except for B/Florida/4/2006 and B/Malaysia/2506/2004.

**Table 6:** Mean values of A/H1N1/California/7/2009-specific Abs and IFN-γ production in study participants who tested positive for H1N1 or coronavirus

	**H1N1 positives**					**Corona positives**					**HC**^**B**^	**HC**^**C**^
	**A**	**B**	**C**	**AB**^**1**^	**AC**^**1**^	**A**	**B**	**C**	**AB**^**1**^	**AC**^**1**^	***p***	
	**SRH (mm**^**2**^**), geometric mean**			***p-value***		**SRH (mm**^**2**^**), geometric mean**			**p-value**			
**A/H1N1/California**	16.1	46.9	30.6	***<0.001***	***0.003***	21.1	29.8	24.3	0.2	0.4	***<0.001***	***0.001***
	**IFN-γ (pg/mL)**											
**A/H1N1/Brisbane**	90	460	274	***<0.001***	***0.01***	156	227	159	0.6	0.2	***<0.001***	***0.01***
**A/H1N1/Solomon**	104	416	272	***<0.001***	***0.01***	124	212	159	0.2	0.2	***<0.01***	***0.03***
**A/H3N2/Uruguay**	125	420	292	***<0.001***	*0.09*	222	224	244	0.8	0.4	***<0.001***	*0.09*
**A/H3N2/Wisconsin**	137	408	298	***<0.001***	***0.02***	212	236	242	0.5	0.4	***0.001***	***0.04***
**A/H5N1/Vietnam**	316	546	392	***<0.01***	*0.3*	355	394	367	0.7	0.8	***<0.01***	*0.3*
**B/Florida**	218	307	327	*0.24*	*0.2*	216	370	240	0.08	0.7	*0.4*	*0.1*
**B/Malaysia**	173	260	251	*0.06*	*0.25*	160	249	177	0.2	0.5	*0.7*	*0.11*
**M1**	34	127	48	***<0.01***	*0.08*	18	24	25	0.04	0.15	*0.07*	*0.25*
**M2**	19	18	19	*0.3*	*0.9*	18	18	18	0.9	0.9	*0.4*	*0.9*
**CFP-10**	19	25	18	*0.9*	*0.3*	18	18	18	0.6	0.3	*0.9*	*0.7*
**PHA**	502	535	588	*0.9*	*0.08*	524	589	544	0.1	0.5	*0.2*	*0.25*
**Mean IFN-γ diff.**	148	265	233			173	220	189				
**N**	21	21	21			38	15	38				

The Wilcoxon-Mann–Whitney rank-sum test was used to calculate the p-values. AB^1^: difference between time point A and B; AC^1^: difference between time point A and C; HC^B^: difference between pdmH1N1 positive and coronavirus positive at time point B. HC^C^ difference between pdmH1N1 positive and coronavirus positive at time point C. Note the strong immune responses at time point B in participants with pdmH1N1 infection; this response was reduced at the end of the study (time point C). P values below 0.05 are indicated in bold.

We observed significantly higher levels of IFN-γ production in response to only 3 flu antigens: A/H3N2/Wisconsin/67/2005, A/H1N1/Brisbane/59/2007 and A/H1N1/Solomon Islands/3/2006 in the flu season (time point C) as compared to the time point zero samples (timepoint A). Thus, the cellular immune response to the H1N1 infection was short-lived, it was directed against a broad panel of flu targets, defined by strong IFN-γ production and pronounced shortly after onset of symptoms (i.e. at the time of the home visit). This was not found to be true several months after the infection: the cellular immune response, defined by IFN-γ production, was weaker and directed only against a narrow panel of flu targets (time point C).

Next, we examined differences between IFN-γ production and A/H1N1/California/7/2009-specific Abs at time points A and C from (i) study participants who received the pdm flu vaccine and reported no ILI symptoms, and from study participants who did not receive the pdm flu vaccine and whose swab results did not test pdmH1N1 PCR positive and (ii) either reported at least one ILI or (iii) reported no ILI symptoms (Table 7).

**Table 7:** Mean values of A/H1N1/California/7/2009-specific Abs and IFN-γ production in blood from study participants with or without pdm flu vaccination and reported either symptoms or no symptoms of ILI

	**Vacc + & Sym+**			**Vacc + & Sym-**			**Vacc- & Sym+**			**Vacc- & Sym-**		
	**A**	**C**	***P***	**A**	**C**	***P***	**A**	**C**	***p***	**A**	**C**	***P***
	**SRH (mm**^**2**^**), geometric mean**											
**A/H1N1/California**	20.6	28.7	***<0.001***	20.4	28.2	**<** ***0.001***	14.8	18.1	*0.4*	20.4	20.7	*0.9*
	**IFN-γ (pg/mL)**											
**A/H1N1/Brisbane**	126	339	***<0.001***	115	279	***<0.001***	156	163	*0.2*	128	158	***0.04***
**A/H1N1/Solomon s/3/2006**	97	293	***<0.001***	90	231	***<0.001***	121	164	*0.2*	108	140	***0.001***
**A/H3N2/Uruguay**	195	350	***<0.001***	162	297	***<0.001***	224	256	*0.3*	170	233	***<0.01***
**A/H3N2/Wisconsin**	207	346	***<0.001***	170	294	***<0.001***	247	264	*0.4*	200	245	***0.04***
**A/H5N1/Vietnam**	346	426	***0.03***	325	427	***<0.001***	336	382	*0.3*	347	363	*0.7*
**B/Florida**	231	316	***0.04***	202	230	*0.06*	280	286	*0.8*	230	267	*0.09*
**B/Malaysia**	138	204	***0.03***	129	163	***<0.01***	191	221	*0.4*	157	183	*0.1*
**M1**	31	81	***<0.01***	27	51	***<0.001***	37	26	*0.3*	30	36	*0.5*
**M2**	18	18	*0.3*	18	20	***0.03***	18	18	*0.3*	18	20	*0.5*
**CFP-10**	21	19	*0.3*	22	24	*0.4*	18	18	*0.9*	21	22	*0.5*
**PHA**	513	521	*0.3*	495	513	*0.05*	500	546	*0.15*	516	534	*0.3*
**Mean IFN-γ**	165	240		151	211		173	199		164	192	
**N**	69	69		312	312		48	48		208	208	

The Wilcoxon-Mann–Whitney rank-sum test was used to calculate the p-values (difference between time point A and B). Note the increased antigen-specific cellular immune responses in blood from participants who did not receive the vaccine and did not report any ILI symptoms. P values below 0.05 are indicated in bold.

We observed significantly increased levels (between time points A and C; p ≤ 0.003) of A/H1N1/California/7/2009-specific Abs in blood from participants who tested positive for H1N1 (and did not receive pdm vaccination; mean increase in Ab titre (SRH): 14.5), and from study participants who received the pdm vaccine (mean increase in Ab titre (SRH): 8). A/H1N1/California/7/2009-specific Abs were above protective levels (>25 mm^2^) for those both groups (vaccinated or non-vaccinated) at time point C.

No significant difference between time points A and C (before and after the flu season) concerning A/H1N1/California/7/2009-specific antibody levels was observed in the group of participants who either tested negative for H1N1 or received flu vaccination (regardless of the reported ILI symptom status); both groups were seropositive but exhibited antibody titres below protective levels.

We observed a very similar IFN-γ production profile in blood from participants who (i) did not receive the pdm flu vaccination, (ii) tested negative for pdmH1N1 and (iii) reported *no* ILI symptoms compared to those who tested H1N1 positive: increased IFN-γ production in response to the flu antigens A/H1N1/Brisbane/59/2007, A/H1N1/Solomon Islands/3/2006, A/H3N2/Wisconsin/67/2005 and A/H3N2/Uruguay/716/2007 (p ≤ 0.04).

## Discussion

This study showed that pdm flu vaccination (A/California/7/2009) induced increased levels of A/H1N1/California/7/2009-specific Abs, but also strong immune cellular responses (measured by IFN-γ production in a whole-blood assay) directed against flu A antigens from H1N1, H3N2 and H5N1 strains, flu B strains, and the flu M1 matrix antigen.

We have not been able to include the pdm flu strain. However, increased IFN-γ production after vaccination suggested that A/California/7/2009-specific T-cell responses were induced by pdm flu vaccination. This would imply that pdm flu vaccine induced (cross)-reactive cellular responses did not only target the flu A H1N1 strains, but also H3N2, H5N1 and flu B strains. In agreement with this observation, we showed that participants who received the pdm flu vaccination exhibited also a significant increase in Ab titers directed against A/H1N1/Solomon Islands/3/2006 and A/H5N1/Vietnam/1203/2004, while participants who did not receive the pdm flu vaccine exhibited only a significant increase in A/H1N1/California/7/2009-specific Abs but not in Abs against the other flu A strains tested by the flu hemagglutination inhibition assay (data not shown).

The flu vaccine, studied in this report, represents a split virus vaccine, which is mainly composed of surface membrane glycoproteins, yet traces of M1 have also been detected [16] as part of the vaccine formulation. We tested M1 and M2 as targets for cellular immune responses in the current study. The M2 protein is a proton-selective ion channel protein, integral in the viral envelope of the influenza A virus. M2 brings protons into the virion core. Acidification of virus interior, leads to weakening of electrostatic interaction and leads to dissociation between M1 (matrix protein) and viral rib nucleoprotein (RNP) complexes [17]. Traces of M1 in the split vaccine may be responsible for the strong anti-M1 directed responses defined by IFN-γ production, which has not been reported until now. Flu vaccines are produced and standardised based on their haemagglutinin and neuraminidase content, and traces of the M1 proteins, contained in the current standard vaccines, may in part be responsible for conferring protective immune responses between flu A strains, since the M1 protein is quite conserved [18]. The M1-directed cellular immune responses, along with traces of M1 proteins in split vaccines may in part responsible for the cross-reactive immune responses against H5N1 associated with the pdm flu vaccine, since CD4+ and CD8+ T-cell responses directed against H5N1 have preferentially reported to focus on M1 or NP (nucleoproteins) [19].

In addition, we showed at study entry that previous seasonal flu vaccination (in 2006–2009) did not lead to an increased IFN-γ production in response to flu antigen components from 2006–2009 flu vaccines, but was instead associated with significantly *lower* IFN-γ production in response to flu antigen components from 2008–2009 flu vaccines (A/H1N1/Brisbane/59/2007, A/H1N1/Solomon Islands/3/2006 and B/Florida/4/2006). A similar trend was observed when analysing H1N1/Brisbane/59/2007- and A/H1N1/Solomon Islands/3/2006-specific antibodies. However, at the end of the study, we could not detect significant differences in IFN-γ production in response to flu antigens between previously vaccinated and unvaccinated participants. This suggests that after the flu season, previously vaccinated and unvaccinated participants were able to mount comparable immune responses (as measured by IFN-γ production in the whole-blood assay).

A recent report showed that individuals with a history of seasonal flu vaccination exhibited after natural pdm flu infection or pdm flu vaccination a skewed Ab response towards previously encountered flu antigens, further proving the impact of previous flu vaccination on subsequent infection or vaccination [20]. Seasonal flu vaccination of children has been reported to interfere with the development of heterosubtypic immunity [21] and Ab responses to pandemic H1N1 appeared to be reduced in participants who received seasonal flu vaccination 3 months prior to vaccination with the pmd flu vaccine [22]. The report by Skowronski and co-workers also suggested an association between the previous 2008–2009 flu vaccination and pandemic H1N1 illness in Canada [23]; the mechanisms underlying this finding are ill-defined and warrant further research to better understand the impact of seasonal flu vaccination, i.e. the potential ‘negative imprint’ of previous vaccinations on cellular immune memory responses.

Reservations about flu vaccination, including the notion that flu vaccination would not result in appropriate protection, have been discussed in the public domain [24-26]. One of the arguments is that flu vaccination may not protect against ILI *symptoms* during flu the season. This notion was corroborated in our study. A number of study participants, after pdm flu vaccination, experienced more ILI symptoms, perhaps due to the observed increase in prevalence of rhinovirus infection in pdm flu vaccinated participants as compared to non-pdm flu vaccinated participants. Other reasons may account for these observations, one of them being the low predictive value of the ILI case definition [26]. Future prospective studies may address the question whether certain flu vaccines are able to increase cellular immune responses in the respiratory system, particularly after encounter with the wildtype flu. Increased ‘influenza-like’ symptoms may occur upon exposure to additional pathogens, such as rhino- or coronavirus, that stimulate ‘flu-primed’ innate or adaptive immune responses. This hypothesis is supported by the observation that infection with rhinovirus may have delayed the circulation of H1N1, most likely via activation of non-specific innate immune responses in the respiratory system [27].

Study participants who received the pdm flu vaccine showed similar IFN-γ production profiles in response to the flu antigens tested, irrespective of the experience of ILI symptoms. However, we observed that study participants who received the pdm flu vaccine and reported ILI symptoms (compared to non-pdm flu vaccinated participants with ILI symptoms), showed by the end of the study a comparable increase in A/California/7/2009-specific Abs and an increase in IFN-γ production in response to the flu A H1N1 and H3N2 strains. This immune status could not be observed in participants who reported no ILI symptoms, irrespective of whether or not they received the pmd flu vaccination. This suggests that some pmd flu vaccinated participants who reported ILI symptoms may have been exposed to the H3N2 seasonal flu supporting previous observations that pmd flu vaccination did not affect the rate of H3N2 infections [28]. The pdmH1N1 strain dominated the flu season in Sweden in 2009–2010, but flu H3N2 and flu B were also present in 1.7% and 1.4% of the samples analysed by the Swedish National Influenza Centre [29].

The comparable increase in A/H1N1/California/7/2009-specific Abs in serum from unvaccinated and pdm flu vaccinated study participants who reported ILI symptoms also suggests that non-pdm flu vaccinated study participants may have been exposed to the pdm flu.

We speculate that the non-pdm flu vaccinated study participants who reported ILI symptoms had been exposed to the pdm flu. These individuals exhibited lower levels of IFN-γ production in response to the H1N1 and H3N2 flu antigens as compared to *pmd flu vaccinated* study participants who reported ILI symptoms. This would also fit with the hypothesis that IFN-γ production directed against related flu antigens upon natural by pdmH1N1 infection is short-lived as compared to IFN-γ induced by pdm flu vaccination. This notion was indeed corroborated: we showed that shortly after a positive pdmH1N1 PCR, IFN-γ production to most of the flu antigens was significantly increased, yet declined by the end of the study (May 2010). In contrast, we could detect significantly higher levels of IFN-γ production in response to M1 (which is not a designated vaccine component) at the end of the study in pdm flu vaccinated participants; this was not the case for study participants who tested positive for pdmH1N1 by PCR (Table 6).

Finally, our observation that study participants i) with a negative pdm flu PCR ii) absent pdm flu vaccination and iii) a negative ILI symptom report, showed increased IFN-γ production in response to most of the flu antigens, concomitant with the absence of A/H1N1/California/7/2009-specific Ab increase, suggests that these individuals may have been exposed to pdm flu, i.e. that they were ‘silently infected’ and developed a strong anti-pdm flu T-cell (but not a B-cell) response.

## Conclusions

The detection of stronger cellular responses measured after the flu season, directed against M1, from study participants who received pdm flu vaccination (as compared to study participants who had a natural pdm flu infection) argues for a more detailed analysis of the role of M1-specific cellular responses induced by vaccination. M1 responses are currently discussed to be crucial in mediating protective anti-flu directed immune responses [30] and may therefore become an important component of future vaccines. The prospective study layout of the LifeGene ILI cohort also demonstrates the value of a time point zero sample to gauge the immune response prior to a vaccination or exposure: pre-existing humoral and cellular immune responses shape the nature of the immune response associated with immune protection or immune pathology.

## Competing interest

The authors declare that they have no competing interest.

## Authors’ contributions

IM was responsible for organization of the study, performance of analyses, data collection and management, data analysis and writing of the manuscript; ME was responsible for statistical analysis and the study design; CL was responsible for coordination of the study, analyses and test performance, RM, LR, AA, RAR, BK, NA, was responsible for patient sample procurement, quality control and assay performance, GP, EM was responsible for the antibody detection assays, AL for epidemiology, NLP for LifeGene’s study design, data analysis and interpretation, MM was responsible for the study design, organization, data analysis, interpretation and writing the manuscript. All authors read and approved the final manuscript.

## Pre-publication history

The pre-publication history for this paper can be accessed here:

http://www.biomedcentral.com/1471-2334/14/319/prepub

## Supplementary Material

Additional file 1: Text Material S1 Describing in detail the recruitment process, surveillance and the PCR-based diagnostics. **Table S1.** Listing the accession number for the respective targets. Click here for file
