# Supplementary material for: Difference in immune response in vaccinated and unvaccinated Swedish individuals after the 2009 influenza pandemic
Source: BMC Infect Dis. 2014 Jun 11;14:319. doi: 10.1186/1471-2334-14-319 (PMC4067073; doi:10.1186/1471-2334-14-319)
Supplement: Additional file 1: Text Material S1 — Describing in detail the recruitment process, surveillance and the PCR-based diagnostics. Table S1. Listing the accession number for the respective targets. [file 1471-2334-14-319-S1.pdf]

Supplementary Information

Supplementary Text Material

S1. Method section

"Influenza-like" illness (ILI) study participants

In September 2009, 2040 study participants from the Stockholm (Sweden) area entered the baseline step of the ILI study in the context of the Swedish national LifeGene project[1]. Participants were recruited mainly at work places (e.g. Biotech companies, Universities, Stockholm Public Transport), and during open-days at LifeGene centers where retirees and children were also invited to join in the study. The participants responded to a baseline web questionnaire addressing existing medical conditions, medication, previous seasonal flu vaccination, physical contact with other people, travelling patterns, physical activity and the intake of supplements. The study participants reported via a web questionnaire (flu event questionnaire) their flu-like symptoms (fever, headache, tiredness, cough, sore throat, runny or stuffy nose, body aches, diarrhoea, vomiting) during the 2009/2010 flu season, and responded to a follow-up web questionnaire (including pandemic flu vaccination status) in spring 2010. Serum and heparin blood samples were drawn at study entry and after the flu season in spring 2010. Prepaid envelopes and nasal swabs were also provided to the participants at study entry and they were invited to use them at the onset of ILI symptoms and send them back for analysis as described below. If the participant's swab was identified with any influenza RNA or corona virus DNA, a home visit (the mean time between the first swab and the home visit was 2,5 weeks) was performed to sample one nasal swab and a blood sample from the index study participant as well as from the household members after they agreed on an informed consent. The study was approved by the regional ethics review board (DN 2009/1183-31) and each individual study participant provided informed consent. Swedish residents who were offered the pandemic flu vaccine received Pandemrix (GSK)

vaccine containing A/ H1N1/California/7/2009 (adjuvanted with ASO3, i.e. DL-α-tocopherol, vitamin E; squalene and polysorbate).

Swab processing

All participants in the study were provided four Σ-VIROCULT swabs and transport media vials (MW950S, Medical Wire and Equipment, Wiltshire, England) during the first blood draw and instructions on how to use the nasal swab. If study participants experienced ILI symptoms, they took a nasal swab and posted the material which was tested for 22 viral targets (Supplementary Table S1) as described in detail[2].

Sample processing and nucleic acid extraction

The swab samples received were blended in the transport medium by vortexing and 400μL of this blend was used to extract viral nucleic acid. Nucleic acid extraction was performed according to supplier's instructions on EZ1 Biorobot (Qiagen Inc. Hilden, Germany) using EZ1 Virus mini kit v2.0 (Qiagen Inc.) and final elution volume of 60μL.

Polymerase chain reaction (PCR) analysis

Virus specific nucleic acid detection was performed using a multiplex kit from Beckman Coulter (Miami, USA) as described in detail[2]. This panel covered 11 different serotypes of Influenza virus and 11 viral serotypes among 6 viruses suspected to cause common upper respiratory tract infections (Supplementary Table S2). Target specific cDNA was synthesized using a reverse transcriptase primer plex kit and for the GenomeLab GeXP start kit (Beckman Coulter). Reverse transcription (RT) reaction was performed in 20μL reaction volume using 12μL of extracted nucleic acid, 4μL of GeXP 5X RT buffer, 2μL of RT primer plex and 1μL each of undiluted Kan-r and reverse transcriptase enzyme. The reaction was initiated at 48°C for 1 minute followed by 42°C for 60 minutes and terminated at 95°C for 5 minutes; the synthesised cDNA was kept at 4°C before using for PCR analysis. PCR reaction was performed in 20μL reaction volume using 9.3μL of synthesized cDNA, 4μL 5X PCR buffer, 4μL, 25mM $MgCl_2$, 2μL PCR primer plex and 0.7μL of Thermo Start DNA polymerase

(Thermo Scientific Inc. Fullerton, CA, USA). The PCR reaction was initiated at 95°C for 5 minutes followed by 40 cycles of 94°C for 30 seconds, 55°C for 30 seconds and 70°C for 1 minute. The PCR product was maintained at 4°C prior to fragment analysis.

PCR fragment analysis and viral target identification

Fragment analysis was performed on a GenomeLab GeXP fragment analysis system (Beckman Coulter Inc.) 1μL of PCR product was dissolved in 39μL of sample loading solution and 400 bp DNA ladder was used as size reference. The Kan-r PCR fragment and RP DNA along with RP RNA PCR fragment were used as reaction control and sample control respectively. If control bands were absent, the analysis was repeated to rule out procedural errors. Viral target identification was performed using GenomeLab GexP fragment analysis software. Once control bands were identified, the sample was subjected to further analysis based on the virus-specific size of the amplicon. In case of influenza A H1N1 09 ('swine flu') serotype, in addition to human housekeeping gene control fragments, the presence of the influenza A generic PCR fragment as well as the H1N1 general signal was required to mark a sample as positive for H1N1 RNA. The detected 'swine flu' positive samples were subjected to sequencing by cloning and subsequent sequencing for matrix (GU322021.1, NCBI), neuraminidase (GU361110.1, NCBI) and hemagglutinin (GU727573.1, NCBI) genes to confirm viral diagnosis.

References

1. Almqvist C, Adami HO, Franks PW, Groop L, Ingelsson E, Kere J, et al. LifeGene--a large prospective population-based study of global relevance. Eur J Epidemiol. 2011; **26**(1): 67-77.
2. Qin M, Wang DY, Huang F, Nie K, Qu M, Wang M, et al. Detection of pandemic influenza A H1N1 virus by multiplex reverse transcription-PCR with a GeXP analyzer. J Virol Methods. 2010; **168**(1-2): 255-8.

Supplementary Tables

Supplementary Table S1. Table of viral targets for the multiplex PCR primer panel.

**Target**	**PCR fragment size (bp)**	**Sequence accession number**
RNAse P (DNA control)	125	NM_006413.4
RNAse P (RNA control)	147	NM_006413.4
Influenza A virus	134	FJ2002421.1
Seasonal H1N1 virus	250	CY037663.1
2009 Novel H1N1 (Swine Flu) virus	236	CQ162202.2
General H1N1 virus	257	CY037663.1
H3N2 (INF A) virus	274	DQ089634.1
H5N1 (INF A) virus	215	EF541394.1
H2N2 (INF A) virus	129	NC_007374
2009 H1 Tamiflu Sensitive	167	GQ117099
2009 H1 Tamiflu Resistant	170	GQ365445
Influenza B virus	204	CY040379.1
Influenza C virus	152	AB000728.1
Adenovirus	140	EF486506
Metapneumovirus	111	AY525843
Parainfluenza 1 virus	149	AF457102
Parainfluenza 2 virus	223	AB367954
Parainfluenza 3 virus	207	FJ455842
Parainfluenza 4 virus	277	M55976
Rhinovirus	160	EF173416
Corona virus-NL63/229E	180	AF304460
Coronavirus HKU1/OC43	287	AY884001
Coronavirus-SARS	240	AY654624
Respiratory syncytial *virus*	243	Z26524
Kan-r (reaction control)	325	-

Supplementary Table S2. Overview of the viral DNA/RNA targets detected by PCR, in nasal swabs from study participants during the flu season 2009/2010.

	N. of virus / swab				
**Virus**	1	2	3	∑	%
Rhinovirus	148	10	1	159	58.9
Corona virus	47	6	.	53	19.6
H1N1 ‘swine flu’	18	8	1	27	10.0
H3N2 flu A	.	3	1	4	1.5
Respiratory syncytial virus	7	1	.	8	3.0
Parainfluenza 1 virus	7	1	.	8	3.0
Influenza C virus	4	.	.	4	1.5
Parainfluenza 2 virus	3	.	.	3	1.1
Metapneumovirus	.	2	.	2	0.7
Parainfluenza 4 virus	1	.	.	1	0.4
Adenovirus	.	1	.	1	0.4
N (viruses)	235	32	3	270	100
N (swabs sent)	235	16	1	252	
N (study participants)	216	16	1	233	
